# Initial blood pressure and adverse cardiac events following acute ischaemic stroke: An individual patient data pooled analysis from the VISTA database

**DOI:** 10.1177/23969873241296391

**Published:** 2024-10-30

**Authors:** Hironori Ishiguchi, Bi Huang, Wahbi K. El-Bouri, Jesse Dawson, Gregory Y. H. Lip, Azmil H. Abdul-Rahim

**Affiliations:** 1 Liverpool Centre for Cardiovascular Science at University of Liverpool, Liverpool John Moores University and Liverpool Heart & Chest Hospital, Liverpool, UK; 2 Department of Medicine and Clinical Science, Yamaguchi University Graduate School of Medicine, Ube, Japan; 3 Department of Cardiovascular and Metabolic Medicine, Institute of Life Course and Medical Sciences, University of Liverpool, Liverpool, UK; 4 School of Cardiovascular and Metabolic Health, College of Medical, Veterinary & Life Sciences, University of Glasgow, Glasgow, UK; 5 Danish Centre for Health Services Research, Department of Clinical Medicine, Aalborg University, Aalborg, Denmark; 6 Stroke Division, Department Medicine for Older People, Mersey and West Lancashire Teaching Hospitals NHS Trust, Prescot, UK

**Keywords:** Ischaemic stroke, stroke-heart syndrome, blood pressure

## Abstract

**Background:**

Adverse cardiac events following ischaemic stroke (stroke-heart syndrome, SHS) pose a clinical challenge. We investigated the association between initial blood pressure at stroke presentation and the risk of SHS.

**Methods:**

We utilised data from the Virtual International Stroke Trials Archive (VISTA). We defined SHS as the incidence of cardiac complications within 30 days post-ischaemic stroke. These presentations included acute coronary syndrome encompassing myocardial injury, heart failure/left ventricular dysfunction, atrial fibrillation/flutter, other arrhythmia/electrocardiogram abnormalities, and cardiorespiratory arrest. Using Cox proportional hazards models, we assessed the risk trajectories for developing SHS and its presentations associated with initial blood pressure. We also explored the risk trajectories for 90-day mortality related to initial blood pressure.

**Results:**

From 16,095 patients with acute ischaemic stroke, 14,965 (mean age 69 ± 12 years; 55% male) were analysed. Of these, 1774 (11.8%) developed SHS. The risk of SHS and initial blood pressure showed a U-shaped relationship. The lowest blood pressures (⩽130 mmHg systolic and ⩽55 mmHg diastolic) were associated with the highest risks (adjusted hazard ratio [95%confidence interval]: 1.40 [1.21–1.63]; *p* < 0.001, 1.71 [1.39–2.10]; *p* < 0.001, respectively, compared to referential blood pressure range).

Cardiorespiratory arrest posed the greatest risk at higher blood pressure levels (2.34 [1.16–4.73]; *p* = 0.017 for systolic blood pressure >190 mmHg), whereas other presentations exhibited the highest risk at lower pressures. The 90-day mortality risk also followed a U-shaped distribution, with greater risks observed at high blood pressure thresholds.

**Conclusions:**

There is a U-shaped relationship between initial blood pressure at ischaemic stroke presentation and the risk of subsequent SHS.

## Graphical abstract

**Graphical abstract:**
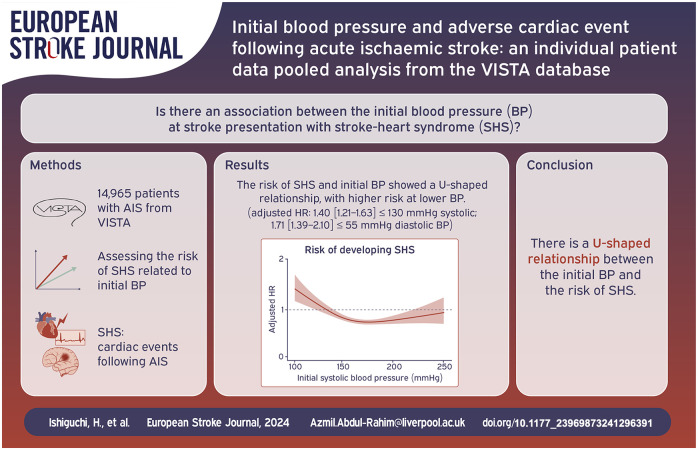

## Introduction

Adverse cardiac events are one of the main post-stroke complications and represent the leading cause of death among non-neurological causes during the acute stroke phase.^1,2^ Furthermore, stroke survivors with cardiac complications typically experience a poor long-term prognosis.^3^

Stroke-heart syndrome (SHS) refers to cardiac complications, either new or worsening of pre-existing heart diseases, occurring within 30 days following the onset of acute ischaemic stroke (AIS).^4,5^ The presentations of SHS include acute myocardial infarction/myocardial injury, heart failure (HF), arrhythmias including electrocardiogram (ECG) abnormalities and atrial fibrillation (AF), and sudden cardiac death.^4,5^ Although SHS exhibits a wide range of presentations, stroke-induced cerebral damage is thought to play a common key role in the development of these complications.^6,7^

The initial blood pressure at presentation is a strong prognostic factor in patients with AIS.^8,9^ The current guidelines recommend avoiding excessive blood pressure (>180/105 mmHg) in patients with AIS undergoing reperfusion therapy, although the effects of aggressive blood-lowering therapy remain controversial.^10^

While most patients typically experience an elevation in blood pressure during the acute phase, a decrease in blood pressure may sometimes occur due to various comorbidities.^9,11^ Previous studies have shown that autonomic dysregulation resulting from cerebral damage influences blood pressure dynamics early in the course of AIS.^8,12^ Since autonomic dysregulation is one of the main triggers for SHS,^6^ there may be relationships between blood pressure variations and the occurrence of adverse cardiac events.

To address this clinical question, we aimed to assess the relationship between initial blood pressure at presentation and the risk of SHS in patients with AIS. In addition, we explored the mortality risk associated with initial blood pressure in patients who developed SHS.

## Methods

### Data resource

We retrospectively analysed individual patient data pooled from randomised clinical trials available within the Virtual International Stroke Trials Archive (VISTA), URL: https://www.virtualtrialsarchives.org/vista/.^13^ VISTA serves as a collaborative platform, collecting patient data from completed acute stroke trials (from the year 1998 to 2010), anonymised in relation to patients and trials’ identity, for novel exploratory analyses. We selected patients with ischaemic stroke who had been randomised to receive placebo or a drug now known to have no confirmed effect on stroke outcomes. The conduct and reporting of our analysis adhered to the Strengthening the Reporting of Observational Studies in Epidemiology guidelines for cohort studies.^14^

### Study design

We extracted data from the VISTA database on patients with AIS, specifically those for whom the information of the initial blood pressure readings at presentation and 90-day mortality data were available. We identified patients who developed SHS, defined as development of at least one of the cardiac complications within 30 days of stroke onset: (1) acute coronary syndrome (ACS) which includes acute myocardial infarction and unstable angina pectoris/myocardial injury (typically referred to as ‘myocardial enzyme elevation’); (2) HF/left ventricular (LV) dysfunction; (3) AF/atrial flutter (AFL); (4) other arrhythmia/ECG abnormalities (e.g. T wave inversion); and (5) cardiorespiratory arrest. We also evaluated the risk trajectories for SHS and each of its presentations relative to the initial blood pressure at stroke presentation. Additionally, we assessed the 90-day mortality risk associated with initial blood pressure in both the total cohort and the SHS cohort.

### Data collection

Two authors (HI and BH) independently reviewed and identified definitive adverse cardiac complications from the list of adverse events in the database. The identified terms were then cross-checked for accuracy and consistency. The initial blood pressure was defined as the single measurement taken at the commencement of acute stroke treatment, coinciding with patient enrolment in each respective trial.

### Statistical analysis

Variables with normal distributions were presented as mean ± standard deviation, and those with non-normal distributions were presented as medians with interquartile ranges (IQR).

We employed two distinct approaches in our analysis using the Cox proportional hazards model. First, we investigated the risk trajectories of SHS and its presentations in relation to the initial systolic and diastolic blood pressure by plotting cubic spline curves. These models were adjusted for six outcome-related with a low missing rate; age, sex, baseline National Institutes of Health Stroke Scale score, use of intravenous thrombolysis, antihypertensive agents and history of AF. The selection of knot numbers for these curves ranged from 3 to 6, optimised for a balance between minimal knots and a lower Akaike Information Criterion.

Second, we performed a stratified analysis, comparing the adjusted hazard ratios (HRs) with 95%confidence interval (CI) for developing SHS across five different intervals of initial systolic and diastolic blood pressure, using the 150–⩽170 mmHg, and 75–⩽95 mmHg interval, respectively as the reference. These reference intervals were chosen because the mean systolic and diastolic blood pressure of the total cohort were fell this range. Similarly, we assessed the risk trajectory for 90-day mortality in relation to initial systolic and diastolic blood pressure among the total cohort and patients with SHS. Missing values were not imputed due to high rates of missing data in several variables, as imputation could potentially introduce bias and results in misleading conclusions. A p-value of less than 0.05 was considered statistically significant. The analyses were conducted using R software, version 4.3.0, on a trusted research network provided by VISTA.

## Results

### Patient demographics

**Figure 1.**
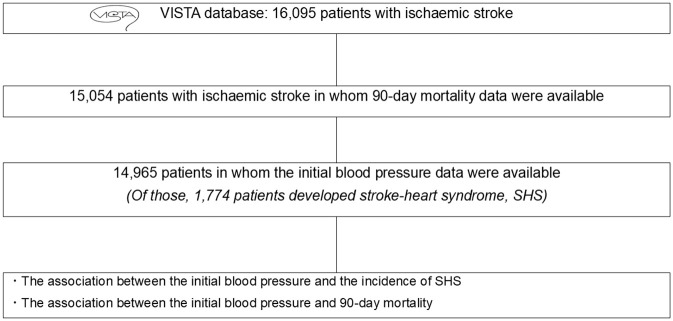
Study diagram. SHS, stroke-heart syndrome; VISTA, Virtual International Stroke Trials Archive.

Figure 1 presents a flow diagram of the study. Out of a total of 16,095 individuals, 14,965 were identified as eligible patients. Among the 6548 terms related to adverse events, 248 were categorised as cardiac events. A total of 1774 patients (12%), corresponding to 2077 adverse events, were recognised as cases of SHS. Patient demographics are outlined in Table 1. The mean systolic/diastolic blood pressure was 157 ± 25 mmHg/85 ± 15 mmHg.

**Table 1.** Patient demographics.

Variables	Patients with acute ischaemic stroke (*n* = 14,965)
Age (years), mean ± SD[0]	69 ± 12
Male, n (%)[0]	8295 (55)
History of stroke, n (%)[0.30]	3293 (22)
Baseline NIHSS, mean ± SD[25]	13 ± 6
Use of IV thrombolysis, n (%)[5.44]	3182 (22)
Use of antihypertensive agents, n (%)[0]	1172 (8)
Systolic blood pressure (mmHg), mean ± SD[0]	157 ± 25
Diastolic blood pressure (mmHg), mean ± SD[0]	85 ± 15
Creatinine (mg/dl), mean ± SD[31]	0.96 ± 0.34
Haemoglobin (g/dl), mean ± SD[37]	14 ± 2
Smoking status[3.50]	
Non, n (%)	6086 (42)
Ex, n (%)	4736 (33)
Current, n (%)	3616 (25)
Comorbidities	
Hypertension, n (%)[5.03]	9645 (68)
Diabetes, n (%)[0.05]	3218 (22)
History of AF, n (%)[14]	3146 (24)
History of MI, n (%)[27]	1466 (13)
History of HF, n (%)[52]	748 (11)
Cardiac events within 30 days from the onset	
Stroke-heart syndrome, n (%)[0]	1774 (12)
ACS/myocardial injury, n (%)[0]	146 (1.0)
HF/LV dysfunction, n (%)[0]	324 (2.2)
AF/AFL, n (%)[0]	602 (4.0)
Other arrhythmia/ECG abnormalities, n (%)[0]	926 (6.2)
Cardiorespiratory arrest, n (%)[0]	79 (0.5)

Numerical data are expressed as mean ± SD or median (interquartile range; first quartile, third quartile). Categorical data are expressed as percentages and numbers. [] indicates missing rates (%).

ACS, acute coronary syndrome; AF, atrial fibrillation; AFL, atrial flutter; ECG, electrocardiogram; HF, heart failure; IV, intravenous; LV, left ventricular; MI, myocardial infarction; NIHSS, National Institutes of Health Stroke Scale; SD, standard deviation; SHS, stroke-heart syndrome.

### The stroke-heart syndrome

Patients developed adverse cardiac events at a median onset time of 2 days (IQR: 1–4) following AIS. The majority of cases were categorised as ‘other arrhythmias/ECG abnormalities’, comprising 52% of total SHS cases (Table 1). Details of the breakdown for each presentation are provided in the Table S1.

The mortality rate in patients with each presentation of SHS was consistently high, reaching 34% across all cases. The SHS presentation associated with the highest mortality was cardiorespiratory arrest, at 91%, while the lowest mortalities were observed in ‘AF/AFL’ and ‘other arrhythmia/ECG abnormalities’ groups, at 28% and 29%, respectively.

### The association between adverse cardiac events and systolic blood pressure

**Figure 2.**
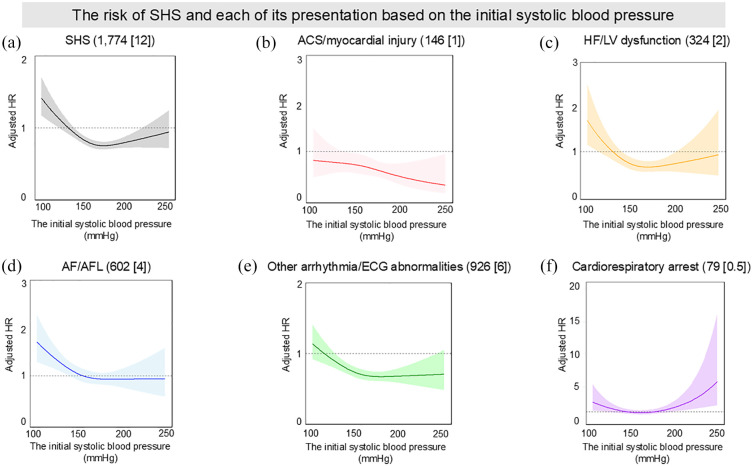
The risk of SHS and each of its presentation based on the initial systolic blood pressure: (a) SHS (n [%]), (b) ACS/myocardial injury (n [%]), (c) HF/LV dysfunction (n [%]), (d) AF/AFL (n [%]), (e) Other arrhythmia/ECG abnormalities (n [%]) and (f) Cardiorespiratory arrest (n [%]). ACS, acute coronary syndrome; AF, atrial fibrillation; AFL, atrial flutter; ECG, electrocardiogram; HF, heart failure; HR, hazard ratio; LV, left ventricular; NIHSS, National Institutes of Health Stroke Scale; SHS, stroke-heart syndrome. Each curve is expressed as adjusted hazard ratio with 95%confidence interval. HRs were adjusted by age, sex, baseline NIHSS, use of intravenous thrombolysis, antihypertensive agents, and history of AF.

Figure 2 displays cubic spline curves illustrating the relationship between initial systolic blood pressure and the risk of developing SHS and its individual presentations. The number of knots for all graphs was set at three, based on the Akaike Information Criterion results (Table S2). The spline curve for total SHS depicted a U-shaped relationship, with risk peaks at approximately 100 mmHg and 250 mmHg (Figure 2(a)). Similarly, all manifestations except for ‘ACS/myocardial injury’ also exhibited a U-shaped risk pattern.

Compared to patients with a systolic blood pressure of > 150 to ⩽170 mmHg, those with ⩽ 130 mmHg were associated with a significantly higher risk of developing SHS (Table S3, adjusted HR [95%CI]: 1.40 [1.21–1.63], *p* < 0.001). As for HF/LV dysfunction, a significantly increased risk was observed at systolic blood pressure levels of ⩽ 130 mmHg and >130 to ⩽150 mmHg (Figure 2(c), adjusted HR [95%CI]: 1.81 [1.22–2.65] and 1.53 [1.09–2.13], respectively). As for ‘AF/AFL’ and ‘other arrhythmia/ECG abnormalities’, the highest risk for developing these conditions was observed at systolic blood pressure levels of ⩽ 130 mmHg. Contrary to these patterns, as for cardiorespiratory arrest, patients displayed the highest risk at > 190 mmHg (adjusted HR [95%CI]: 2.34 [1.16–4.73], *p* = 0.017), highlighting a distinct risk profile in this presentation (Figure 2(f)).

### Associations between adverse cardiac events and diastolic blood pressure

**Figure 3.**
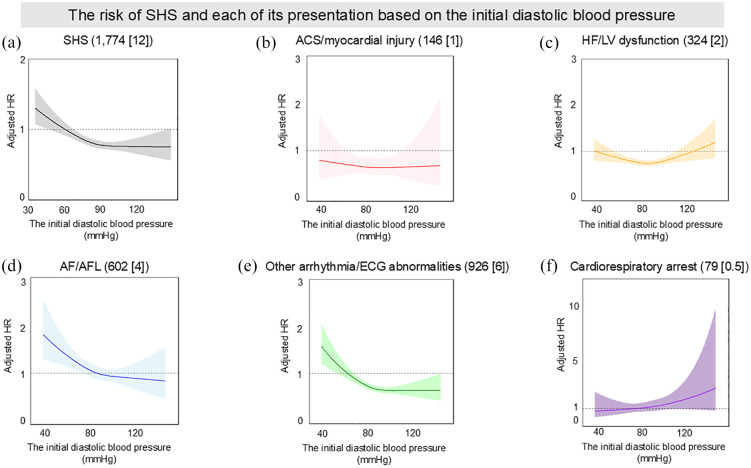
The risk of SHS and each of its presentation based on the initial diastolic blood pressure: (a) SHS (n [%]), (b) ACS/myocardial injury (n [%]), (c) HF/LV dysfunction (n [%]), (d) AF/AFL (n [%]), (e) Other arrhythmia/ECG abnormalities (n [%]) and (f) Cardiorespiratory arrest (n [%]). ACS, acute coronary syndrome; AF, atrial fibrillation; AFL, atrial flutter; ECG, electrocardiogram; HF, heart failure; HR, hazard ratio; LV, left ventricular; NIHSS, National Institutes of Health Stroke Scale; SHS, stroke-heart syndrome. Each curve is expressed as adjusted hazard ratio with 95%confidence interval. HRs were adjusted by age, sex, baseline NIHSS, use of intravenous thrombolysis, and history of AF.

Figure 3 shows cubic spline curves illustrating the relationship between initial diastolic blood pressure and the risk of developing SHS. Consistent with the analysis in systolic blood pressure, the number of knots in each presentation was set at three. The relationship for SHS development also showed a U-shaped curve, with risk peaks at lower blood pressures (Figure 3(a)). Notably, diastolic blood pressure ranges of ⩽ 55 mmHg and > 55–⩽75 mmHg were associated with significantly higher risks of SHS compared to the reference range of > 75 to ⩽95 mmHg (Table S4). The SHS presentations of ‘ACS/myocardial injury’, ‘HF/LV dysfunction’, and ‘other arrhythmia/ECG abnormalities’ each displayed similar U-shaped risk profiles, peaking at lower pressures. In contrast, AF/AFL displayed an L-shaped curve, particularly peaking at lower blood pressures (Figure 3(d)). Similarly, cardiorespiratory arrest demonstrated an L-shaped curve, with a pronounced increase in risk at higher blood pressures (Figure 3(f)).

### Associations between mortality risk and blood pressure

**Figure 4.**
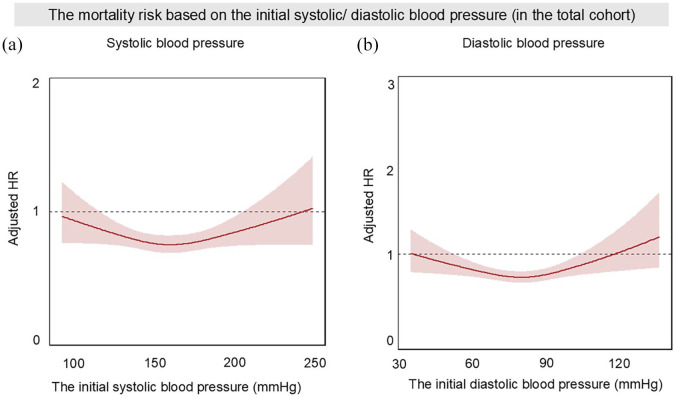
The risk of 90-day mortality based on the initial systolic and diastolic blood pressure within the total cohort: (a) The risk trajectory for systolic blood pressure and (b) The risk trajectory for diastolic blood pressure. AF, atrial fibrillation; HR, hazard ratio; NIHSS, National Institutes of Health Stroke Scale. Each curve is expressed as adjusted hazard ratio with 95%confidence interval. HRs were adjusted by age, sex, baseline NIHSS, use of intravenous thrombolysis, antihypertensive agents, and history of AF.

Figure 4 presents cubic spline curves illustrating the relationship between initial systolic/diastolic blood pressures and the 90-day mortality risk in the total cohort. For both measurements, the numbers of knots were set at three. Both systolic and diastolic blood pressure exhibited similar U-shaped trends, analogous to the risk trajectory for SHS development. However, their dynamics differed, with peak risk observed at high blood pressure levels.

Regarding systolic blood pressure, patients with levels exceeding 190 mmHg exhibited an increased risk of mortality compared to those in the > 150 to ⩽170 mmHg range (Table S5). Concerning diastolic blood pressure, both higher (>115 mmHg) and lower (⩽55 mmHg) pressures were associated with significantly increased mortality risks (Table S5, adjusted HR [95%CI]: 1.43 [1.04–1.97] for > 115 mmHg and 1.32 [1.02–1.72] for ⩽ 55 mmHg, respectively).

In patients with SHS, both systolic and diastolic blood pressure also mirrored the U-shaped trend observed in the total cohort, with the peak occurring at higher levels, as shown in Figure S1.

For systolic blood pressure, a notable escalation in risk was observed at elevated levels (Figure S1A). Specifically, patients with a systolic blood pressure exceeding 190 mmHg were associated with a significantly increased risk of mortality compared to those within the > 150 to ⩽170 mmHg range (Table S6, adjusted HR [95%CI]: 1.46 [1.05–2.04], *p* = 0.022).

Conversely, while the diastolic blood pressure also displayed a U-shaped curve peaking at higher pressures, the contrast in risk between lower and higher pressures was less pronounced (Figure S1B). Patients with diastolic pressures > 115 mmHg showed a higher, but not statistically significant, mortality risk compared to those within the > 75 to ⩽95 mmHg range. This was followed closely by those at ⩽ 55 mmHg (Table S6, >115 mmHg: adjusted HR [95%CI]: 1.50 [0.87–2.59], *p* = 0.141 ; ⩽55 mmHg: 1.43 [0.99–2.05], *p* = 0.052).

## Discussion

The key findings of this study are summarised as follows (Visual Abstract): First, the relationship between the initial systolic and diastolic blood pressure and the risk of SHS is U-shaped. For both measurements, the lowest blood pressures (⩽130 mmHg systolic and ⩽55 mmHg diastolic) are associated with the highest risks. Second, specific SHS presentations such as ‘HF/LV dysfunction’, ‘AF/AFL’, and ‘other arrhythmia/ECG abnormalities’ generally present the highest risks at lower systolic and diastolic blood pressures. Conversely, cardiorespiratory arrest demonstrates a distinctly different profile, showing the greatest risk at higher blood pressures. ‘ACS/myocardial injury’ exhibits the highest risk, as quantified by adjusted HR, specifically at lower diastolic blood pressures, showing no similar trend at systolic blood pressures. Third, mortality risk within 90 days for patients with SHS also follows a U-shaped distribution for both systolic and diastolic pressures. However, the most pronounced mortality risks are observed at higher pressure thresholds (>190 mmHg systolic and >115 mmHg diastolic).

### Blood pressure in ischaemic stroke

Previous studies have indicated that the relationship between initial blood pressure and prognosis in stroke patients is U-shaped: increased risks of mortality, stroke recurrence, and dependency were observed at both higher and lower ends of the systolic and diastolic blood pressure spectrum.^9,15,16^ However, the mechanisms driving increased risks at high and low blood pressure extremes are believed to be distinct. In cases of high blood pressure, excessive sympathetic activity, cerebral oedema, elevated intracranial pressure resulting from brain injury are implicated in both the elevation of blood pressure and the deterioration of prognosis.^8,12^ Of note, the sympathetic overactivity, typically induced by direct injury to modulatory brain regions and impaired sensitivity of cardiac baroreceptors, can trigger various malignant pathways, including elevated levels of circulating catecholamines and stimulation of inflammatory pathways, leading to systemic organ failure and cardiac events.^7,8^ In contrast, low blood pressure is considered to derive from non-neurological conditions. Potential contributors to low blood pressure involve the hypovolaemia, typically resulting from haemorrhage, systemic inflammatory response syndrome, and cardiac co-morbidities that limit cardiac output.^11^ Indeed, patients with AIS presenting with low blood pressure tended to have cardiac comorbidities such as a history of myocardial infarction and HF, and they faced an increased risk of developing HF during the acute phase.^15,16^

In line with previous findings, we have newly demonstrated that the U-shaped relationship previously observed in blood pressure among patients with AIS is also evident in the development of adverse cardiac complications. While most cardiac presentations exhibit a U-shaped relationship with blood pressure, the highest risk levels differ depending on the specific SHS presentations. Specifically, arrhythmia, HF/LV dysfunction and ACS/myocardial injury present the highest risk at lower blood pressure levels, whereas the risk for cardiorespiratory arrest peaks at higher levels.

Several explanations account for this disparity. First, the higher incidence of cardiac events at lower blood pressure levels may reflect an exacerbation of pre-existing cardiac comorbidities that limit cardiac output. This inference is supported by previous literature, which reports that low blood pressure indicates the worsening of underlying cardiac conditions.^15,16^ Additionally, low blood pressure itself can exacerbate myocardial perfusion, contributing causally to the development of more cardiac complications. Notably, studies in general hypertensive populations have shown that low diastolic blood pressure makes individuals more susceptible to cardiovascular events, including coronary events, likely due to the worsening of coronary perfusion.^17,18^ Consistent with these findings, our results also demonstrate that the risk of ACS/myocardial injury tended to elevate at lower diastolic blood pressure, but not systolic blood pressure. Our analysis also revealed a discrepancy in the risk of ACS/myocardial injury between systolic and diastolic blood pressure in the higher blood pressure ranges. While systolic blood pressure showed a gradual decrease in risk with increasing pressure, diastolic blood pressure did not follow this trend in higher ranges. This difference may be due to the blood pressure distribution in our study population, where cases of high diastolic blood pressure were considerably fewer than those with high systolic blood pressure (systolic blood pressure > 190 mmHg: 11% vs diastolic blood pressure > 115 mmHg: 3% of the total population). The limited number of ACS/myocardial injury cases (1% of the total population) is also likely to compound this effect. This interpretation is supported by the wider 95%CI for the adjusted hazard ratio in high diastolic blood pressure (>115 mmHg: 0.53 [0.13–2.21]) compared to high systolic blood pressure (>190 mmHg: 0.71 [0.35–1.44]). Consequently, the generalisability of our findings may be limited. Further studies in populations with a higher prevalence of elevated diastolic blood pressure are warranted to clarify these relationships and validate our findings.

As for the risks of adverse cardiac events associated with higher blood pressure, we postulate that sympathetic overactivity is likely the primary culprit. The sympathetic overactivity is considered as one of the pivotal mechanisms for developing cardiac events following a stroke.^7^ In particular, we infer that cardiorespiratory arrest primarily contributed to this observation, given the risk trajectory of this presentation showed a particularly marked increase at higher blood pressure levels. The cardiorespiratory arrest may be triggered by sympathetic overactivity, as literature using experimental models suggested that sympathetic overactivity not only elevates blood pressure but also plays a pivotal role in sudden cardiac death.^19^ The pro-sympathetic state is particularly pronounced in strokes involving the insular cortex, which have been strongly suggested to link to sudden cardiac death.^20,21^ However, our study did not include information on the anatomical stroke location, thus precluding an analysis of the extent of insular involvement in the patients with high blood pressure.

### Mortality risk in relation to blood pressure in patients with SHS

We demonstrated a U-shaped relationship between systolic/diastolic blood pressure and 90-day mortality risk in patients with SHS, mirroring the relationship between blood pressure and adverse cardiac event risks. Although the relationship is U-shaped, the peak risk occurs at higher blood pressure levels. We postulate that this finding reflects that individuals at both extremes of the blood pressure spectrum—higher and lower—experience distinct types of risk manifestations. Specifically, we believe that the elevated mortality risk in individuals with higher blood pressure is primarily due to their increased risk of cardiorespiratory arrest, which carries a higher mortality risk compared to other SHS presentations. On the other hand, the elevated mortality risk in lower blood pressure would be due to other presentations, especially HF/LV dysfunction and arrhythmias, although not as high as the mortality risks associated with cardiorespiratory arrest. Our data suggest that higher and lower blood pressures similarly escalate the risks of other SHS presentations, contributing to increased mortality rates.

### Clinical implications

Our findings have important clinical implications for the management of patients with AIS. We have demonstrated a U-shaped relationship between the initial blood pressure measurements and the incidence of SHS, as well as mortality risk. Notably, our data suggest that the risk of specific SHS presentations varies across the blood pressure spectrum. Arrhythmias, including AF/AFL, and HF/LV dysfunction demonstrate the highest risk at lower systolic/diastolic blood pressures. In contrast, the risk of cardiorespiratory arrest was greatest at higher blood pressure levels. For mortality, while the relationship follows a U-shaped pattern, the risk is more pronounced at higher blood pressure levels.

These observations provide clinicians with a valuable tool for risk assessment in patients with AIS. Evaluation of the initial blood pressure measurements may offer prognostic information on the risk of specific SHS presentations and overall mortality risk, potentially guiding more targeted monitoring and early intervention strategies.

### Limitations

Our study has several limitations. First, the identification of cardiac events was dependent on the documentation of adverse events reporting across various clinical trials included in the VISTA database, rather than direct examination of medical records. In addition, the smaller number of cardiorespiratory arrest events raises the possibility that our findings related to this outcome could be due to chance rather than representing a true association. This approach may lead to underestimation of the true incidence of SHS, particularly in asymptomatic patients. Second, our cohort primarily consisted of individuals for whom high-sensitivity troponin assays were unavailable. Consequently, up to 40% of patients might have been diagnosed with acute myocardial injury if the assay had been implemented.^22^ Therefore, the incidence of this SHS presentation could differ from that in the current population. It is also important to note that our data were pooled from historical clinical trials conducted between 1998 and 2010. Given the many advancements in stroke management since then, our results may have limited generalisability to current practice. Third, our study design, which focuses on a single blood pressure measurement, cannot evaluate cases that experienced multiple phases of blood pressure changes. Moreover, the database does not include information regarding the patient’s position during measurement or specific details about the blood pressure measurement procedure. Hence, we are unable to address the relationship between cardiac events and blood pressure variability, which impacts both short- and long-term prognosis and dependency in patients with AIS, regardless of therapeutic strategies.^23^ In addition, lower blood pressure in patients with AIS can reflect a worsening systemic condition. Since sympathetic nerve overactivity can be involved in the development of any cardiac events,^7^ we cannot exclude the possibility that patients who experienced SHS other than cardiopulmonary arrest, also underwent a phase of elevated blood pressure before it decreased due to their deteriorating general condition. Fourth, our population predominantly consisted of patients with moderate to severe stroke, as indicated by the high mean baseline NIHSS score (13 ± 6). This skew towards more severe cases limits the generalisability of our findings to the broader stroke population, particularly those with minor strokes. Fifth, the VISTA database primarily includes data from previous clinical trials, which may not fully represent the general population of patients with AIS. Future studies that incorporate more diverse patient populations, particularly those typically excluded from trials, would be valuable to confirm the generalisability of our findings. Finally, our study lacks information on the blood pressure inclusion/exclusion criteria from the original trials. This may introduce selection bias, which could potentially influence our findings and should be considered when interpreting the results.

## Conclusions

There is a U-shaped relationship between initial blood pressure at stroke presentation and the risk of subsequent SHS.

## Supplemental Material

sj-docx-1-eso-10.1177_23969873241296391 – Supplemental material for Initial blood pressure and adverse cardiac events following acute ischaemic stroke: An individual patient data pooled analysis from the VISTA database Supplemental material, sj-docx-1-eso-10.1177_23969873241296391 for Initial blood pressure and adverse cardiac events following acute ischaemic stroke: An individual patient data pooled analysis from the VISTA database by Hironori Ishiguchi, Bi Huang, Wahbi K. El-Bouri, Jesse Dawson, Gregory Y. H. Lip and Azmil H. Abdul-Rahim in European Stroke Journal
