# Supplementary material for: Initial blood pressure and adverse cardiac events following acute ischaemic stroke: An individual patient data pooled analysis from the VISTA database
Source: Eur Stroke J. 2024 Oct 30;10(2):469–77. doi: 10.1177/23969873241296391 (PMC11556537; doi:10.1177/23969873241296391)
Supplement: sj-docx-1-eso-10.1177_23969873241296391 – Supplemental material for Initial blood pressure and adverse cardiac events following acute ischaemic stroke: An individual patient data pooled analysis from the VISTA database [file sj-docx-1-eso-10.1177_23969873241296391.docx]

**Supplementary Materials**

**Table S1:** Detailed Breakdown of Each Presentation

**Table S2:** Comparison of Akaike Information Criterion Values for Different Number of Knots in Spline Curves (Risk of Cardiac Events)

**Table S3:** Comparison of the Risk of Adverse Cardiac Events Across the Initial Systolic Blood Pressure

**Table S4:** Comparison of the Risk of Adverse Cardiac Events Across the Initial Diastolic Blood Pressure

**Table S5:** Comparison of the 90-day Mortality Risks Across the Initial Systolic/Diastolic Blood Pressure in Total Cohort

**Table S6:** Comparison of the 90-day Mortality Risks Across the Initial Systolic/Diastolic Blood Pressure within Patients with Stroke-Heart Syndrome

**Figure S1:** The Risk of 90-day Mortality Based on the Initial Systolic and Diastolic Blood Pressure within the SHS Cohort

**Table S1. Detailed Breakdown of Each Presentation**

| **ACS/myocardial injury (n=146)** | |
| --- | --- |
| ACS, n (%) | 133 (91) |
| Myocardial injury, n (%) | 13 (9) |
| **HF/LV dysfunction (n=325)** | |
| HF, n (%) | 298 (92) |
| LV dysfunction, n (%) | 27 (8) |
| **AF/AFL (n=602)** | |
| AF, n (%) | 566 (94) |
| AFL, n (%) | 36 (6) |
| **Other arrhythmia/ECG abnormalities (n=926)** | |
| Other arrhythmia, n (%) | 875 (94) |
| ECG abnormalities, n (%) | 51 (6) |

ACS, acute coronary syndrome; AF, atrial fibrillation; AFL, atrial flutter; ECG, electrocardiogram; HF, heart failure; LV, left ventricular

**Table S2. Comparison of Akaike Information Criterion Values for Different Number of Knots in Spline Curves (Risk of Cardiac Events)**

|  | Knot | | | |
| --- | --- | --- | --- | --- |
|  | 3 | 4 | 5 | 6 |
| **Risk for SHS in Systolic Blood Pressure** | | | | |
| SHS | 30,219 | 30,221 | 20,221 | 30,223 |
| ACS/myocardial injury | 2,161 | 2,163 | 2,164 | 2,166 |
| HF/LV dysfunction | 4,442 | 4,441 | 4,443 | 4,444 |
| AF/AFL | 9,378 | 9,379 | 9,381 | 9,383 |
| Other arrhythmia/ECG abnormalities | 15,162 | 15,163 | 15,164 | 15,165 |
| Cardiorespiratory arrest | 1,381 | 1,383 | 1,385 | 1,386 |
| **Risk for SHS in Diastolic Blood Pressure** | | | | |
| SHS | 30,227 | 30,230 | 30,225 | 30,226 |
| ACS/ myocardial injury | 2,170 | 2,171 | 2,172 | 2,173 |
| HF/LV dysfunction | 4,458 | 4,459 | 4,460 | 4,461 |
| AF/AFL | 9,379 | 9,380 | 9,382 | 9,383 |
| Other arrhythmia/ECG abnormalities | 15,144 | 15,145 | 15,138 | 15,139 |
| Cardiorespiratory arrest | 1,389 | 1,390 | 1,392 | 1,393 |
| **Risk for Ninety-Day Mortality in Systolic Blood Pressure** | | | | |
| Total cohort | 20,023 | 20,025 | 20,027 | 20,028 |
| SHS | 6,359 | 6,361 | 6,361 | 6,363 |
| **Risk for Ninety-Day Mortality in Diastolic Blood Pressure** | | | | |
| Total cohort | 20,019 | 20,021 | 20,022 | 20,024 |
| SHS | 6,376 | 6,377 | 6,376 | 6,377 |

ACS, acute coronary syndrome; AF, atrial fibrillation, AFL, atrial flutter; ECG, electrocardiogram; HF, heart failure; LV, left ventricle; SHS, stroke-heart syndrome

**Table S3. Comparison of the Risk of Adverse Cardiac Events Across the Initial Systolic Blood Pressure**

|  | Adjusted HR (95%CI) | P-value |
| --- | --- | --- |
| SHS, (n [%]) | | |
| ≤130 mmHg, (1,662 [11])* | 1.40 (1.21-1.63) | <0.001 |
| >130 to ≤150 mmHg, (3,892 [26]) | 1.10 (0.97-1.25) | 0.122 |
| >150 to ≤170 mmHg, (4,762 [32]) | 1.00 (Reference) |  |
| >170 to ≤190 mmHg, (3,064 [20]) | 0.97 (0.83-1.12) | 0.684 |
| >190 mmHg, (1,585 [11]) | 0.99 (0.83-1.18) | 0.965 |
| ACS/myocardial injury | | |
| ≤130 mmHg | 1.07 (0.61-1.90) | 0.792 |
| >130 to ≤150 mmHg | 1.07 (0.67-1.70) | 0.768 |
| >150 to ≤170 mmHg | 1.00 (Reference) |  |
| >170 to ≤190 mmHg | 0.75 (0.43-1.31) | 0.316 |
| >190 mmHg | 0.71 (0.35-1.44) | 0.353 |
| HF/LV dysfunction | | |
| ≤130 mmHg * | 1.81 (1.22-2.65) | 0.002 |
| >130 to ≤150 mmHg * | 1.53 (1.09-2.13) | 0.011 |
| >150 to ≤170 mmHg | 1.00 (Reference) |  |
| >170 to ≤190 mmHg | 0.97 (0.65-1.44) | 0.889 |
| >190 mmHg | 1.02 (0.63-1.66) | 0.915 |
| AF/AFL | | |
| ≤130 mmHg * | 1.58 (1.22-2.05) | <0.001 |
| >130 to ≤150 mmHg | 1.15 (0.91-1.45) | 0.225 |
| >150 to ≤170 mmHg | 1.00 (Reference) |  |
| >170 to ≤190 mmHg | 1.11 (0.86-1.43) | 0.389 |
| >190 mmHg | 0.76 (0.53-1.08) | 0.129 |
| Other arrhythmia/ECG abnormalities | | |
| ≤130 mmHg * | 1.44 (1.17-1.77) | <0.001 |
| >130 to ≤150 mmHg | 1.14 (0.95-1.38) | 0.134 |
| >150 to ≤170 mmHg | 1.00 (Reference) |  |
| >170 to ≤190 mmHg | 1.02 (0.83-1.25) | 0.835 |
| >190 mmHg | 0.91 (0.70-1.18) | 0.493 |
| Cardiorespiratory arrest | | |
| ≤130 mmHg | 1.52 (0.72-3.20) | 0.267 |
| >130 to ≤150 mmHg | 1.41 (0.74-2.66) | 0.288 |
| >150 to ≤170 mmHg | 1.00 (Reference) |  |
| >170 to ≤190 mmHg | 1.06 (0.50-2.23) | 0.836 |
| >190 mmHg * | 2.34 (1.16-4.73) | 0.017 |

HRs were adjusted by age, sex, baseline NIHSS, use of intravenous thrombolysis antihypertensive agents, and history of AF. Asterisk (*) indicates statistical significance (P<0.05).

ACS, acute coronary syndrome; AF, atrial fibrillation, AFL, atrial flutter; CI, confidence interval; ECG, electrocardiogram; HF, heart failure; HR, hazard ratio; LV, left ventricular; NIHSS, National Institutes of Health Stroke Scale; SHS, stroke-heart syndrome

**Table S4. Comparison of the Risk of Adverse Cardiac Events Across the Initial Diastolic Blood Pressure**

|  | Adjusted HR (95%CI) | P-value |
| --- | --- | --- |
| SHS, (n [%]) | | |
| ≤55 mmHg, (371 [2])* | 1.71 (1.39-2.10) | <0.001 |
| >55 to ≤75 mmHg, (3,265 [22])* | 1.26 (1.12-1.41) | <0.001 |
| >75 to ≤95 mmHg, (7,527 [50]) | 1.00 (Reference) |  |
| >95 to ≤115 mmHg, (3,388 [23]) | 1.11 (0.97-1.26) | 0.118 |
| >115 mmHg, (414 [3]) | 0.83 (0.59-1.16) | 0.291 |
| ACS/myocardial injury | | |
| ≤55 mmHg | 1.78 (0.84-3.76) | 0.128 |
| >55 to ≤75 mmHg | 0.79 (0.49-1.26) | 0.330 |
| >75 to ≤95 mmHg | 1.00 (Reference) |  |
| >95 to ≤115 mmHg | 1.04 (0.65-1.66) | 0.854 |
| >115 mmHg | 0.53 (0.13-2.21) | 0.391 |
| HF/LV dysfunction | | |
| ≤55 mmHg | 1.48 (0.86-2.56) | 0.155 |
| >55 to ≤75 mmHg | 1.17 (0.86-1.58) | 0.299 |
| >75 to ≤95 mmHg | 1.00 (Reference) |  |
| >95 to ≤115 mmHg | 1.24 (0.89-1.74) | 0.197 |
| >115 mmHg | 1.28 (0.62-2.64) | 0.497 |
| AF/AFL | | |
| ≤55 mmHg * | 1.65 (1.16-2.36) | 0.005 |
| >55 to ≤75 mmHg * | 1.33 (1.09-1.62) | 0.004 |
| >75 to ≤95 mmHg | 1.00 (Reference) |  |
| >95 to ≤115 mmHg | 0.90 (0.69-1.17) | 0.449 |
| >115 mmHg | 1.08 (0.61-1.90) | 0.774 |
| Other arrhythmia/ECG abnormalities | | |
| ≤55 mmHg * | 2.41 (1.84-3.16) | <0.001 |
| >55 to ≤75 mmHg * | 1.48 (1.26-1.74) | <0.001 |
| >75 to ≤95 mmHg | 1.00 (Reference) |  |
| >95 to ≤115 mmHg * | 1.20 (1.00-1.45) | 0.049 |
| >115 mmHg | 0.71 (0.42-1.19) | 0.198 |
| Cardiorespiratory arrest | | |
| ≤55 mmHg | 0.61 (0.14-2.56) | 0.505 |
| >55 to ≤75 mmHg | 0.76 (0.42-1.38) | 0.384 |
| >75 to ≤95 mmHg | 1.00 (Reference) |  |
| >95 to ≤115 mmHg | 1.20 (0.68-2.11) | 0.525 |
| >115 mmHg | 2.24 (0.87-5.76) | 0.093 |

HRs were adjusted by age, sex, baseline NIHSS, use of intravenous thrombolysis, antihypertensive agents, and history of AF. Asterisk (*) indicates statistical significance (P<0.05).

ACS, acute coronary syndrome; AF, atrial fibrillation, AFL, atrial flutter; CI, confidence interval; ECG, electrocardiogram; HF, heart failure; HR, hazard ratio; LV, left ventricular; NIHSS, National Institutes of Health Stroke Scale; SHS, stroke-heart syndrome

**Table S5. Comparison of the 90-day Mortality Risks Across the Initial Systolic/Diastolic Blood Pressure in Total Cohort**

|  | Adjusted HR (95%CI) | P-value |
| --- | --- | --- |
| The total cohort (N=14,965) | | |
| Initial systolic blood pressure, (n [%]) | | |
| ≤130 mmHg, (1,662 [11]) | 1.02 (0.84-1.24) | 0.781 |
| >130 to ≤150 mmHg, (3,892 [26]) | 1.06 (0.91-1.24) | 0.418 |
| >150 to ≤170 mmHg, (4,762 [32]) | 1.00 (Reference) |  |
| >170 to ≤190 mmHg, (3,064 [20]) | 1.05 (0.88-1.24) | 0.562 |
| >190 mmHg, (1,585 [11]) | 1.11 (0.91-1.36) | 0.288 |
| Initial diastolic blood pressure | | |
| ≤55 mmHg, (371 [2])* | 1.32 (1.02-1.72) | 0.033 |
| >55 to ≤75 mmHg, (3,265 [22]) | 1.10 (0.95-1.26) | 0.179 |
| >75 to ≤95 mmHg, (7,527 [50]) | 1.00 (Reference) |  |
| >95 to ≤115 mmHg, (3,388 [23]) | 1.09 (0.93-1.28) | 0.271 |
| >115 mmHg, (414 [3])* | 1.43 (1.04-1.97) | 0.024 |

HRs were adjusted by age, sex, baseline NIHSS, use of intravenous thrombolysis, antihypertensive agents, and history of AF. Asterisk (*) indicates statistical significance (P<0.05).

AF, atrial fibrillation; HR, hazard ratio; NIHSS, National Institutes of Health Stroke Scale.

**Table S6. Comparison of the 90-day Mortality Risks Across the Initial Systolic/Diastolic Blood Pressure within Patients with Stroke-Heart Syndrome**

|  | Adjusted HR (95%CI) | P-value |
| --- | --- | --- |
| Stroke-heart syndrome (N=1,774) | | |
| Initial systolic blood pressure | | |
| ≤130 mmHg, (306 [17]) | 1.32 (0.99-1.78) | 0.057 |
| >130 to ≤150 mmHg, (479 [27]) | 1.12 (0.86-1.45) | 0.382 |
| >150 to ≤170 mmHg, (488 [28]) | 1.00 (Reference) |  |
| >170 to ≤190 mmHg, (320 [18]) | 1.21 (0.92-1.60) | 0.158 |
| >190 mmHg, (181 [10]) * | 1.46 (1.05-2.04) | 0.022 |
| Initial diastolic blood pressure | | |
| ≤55 mmHg, (108 [6]) | 1.43 (0.99-2.05) | 0.052 |
| >55 to ≤75 mmHg, (524 [30]) | 0.98 (0.78-1.23) | 0.896 |
| >75 to ≤95 mmHg, (762 [43]) | 1.00 (Reference) |  |
| >95 to ≤115 mmHg, (344 [19]) | 1.04 (0.80-1.36) | 0.737 |
| >115 mmHg, (36 [2]) | 1.50 (0.87-2.59) | 0.141 |

HRs were adjusted by age, sex, baseline NIHSS, use of intravenous thrombolysis, antihypertensive agents, and history of AF. Asterisk (*) indicates statistical significance (P<0.05).

AF, atrial fibrillation; HR, hazard ratio; NIHSS, National Institutes of Health Stroke Scale

**Figure S1. The Risk of 90-day Mortality Based on the Initial Systolic and Diastolic Blood Pressure within the SHS Cohort**
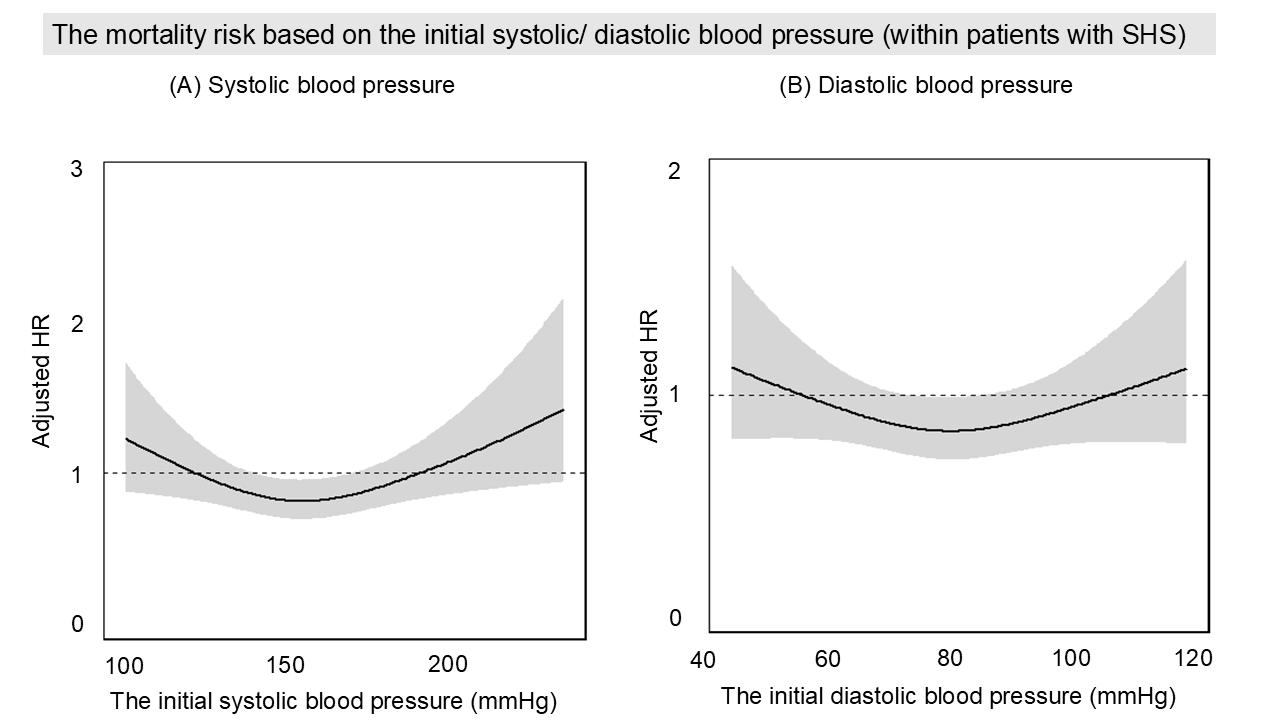


(A) The risk trajectory for systolic blood pressure. (B) The risk trajectory for diastolic blood pressure. AF, atrial fibrillation; HR, hazard ratio; NIHSS, National Institutes of Health Stroke Scale; SHS, stroke-heart syndrome. Each curve is expressed as adjusted hazard ratio with 95% confidence interval. HRs were adjusted by age, sex, baseline NIHSS, use of intravenous thrombolysis, and history of AF.
